# Clinical research diagnostic criteria for bipolar illness (CRDC-BP): rationale and validity

**DOI:** 10.1186/s40345-022-00267-3

**Published:** 2022-10-13

**Authors:** S. Nassir Ghaemi, Jules Angst, Paul A. Vohringer, Eric A. Youngstrom, James Phelps, Philip B. Mitchell, Roger S. McIntyre, Michael Bauer, Eduard Vieta, Samuel Gershon

**Affiliations:** 1 Department of Psychiatry, Tufts University, 800 Washington St, Boston, MA 02111 USA; 2 Department of Psychiatry, Harvard Medical School, Boston, USA; 3 Zurich University, Zurich, Switzerland; 4 Department of Psychiatry, University of Chile, Santiago, Chile; 5 Departments of Psychology, Neuroscience, and Psychiatry, University of North Carolina, Chapel Hill, NC USA; 6 Department of Psychiatry, Good Samaritan Regional Medical Center, Corvallis, OR USA; 7 Discipline of Psychiatry and Mental Health, University of New South Wales, Sydney, Australia; 8 Department of Psychiatry, University of Toronto, Toronto, Canada; 9 Department of Psychiatry and Psychotherapy, Carl Gustav Carus University Hospital, Technische Universität Dresden, Dresden, Germany; 10 Department of Psychiatry and Psychology, Hospital Clinic, Institute of Neuroscience, University of Barcelona, IDIBAPS, CIBERSAM, Barcelona, Catalonia Spain; 11 Department of Psychiatry, University of Miami, Miami, USA

**Keywords:** DSM, RDC, CRDC, Diagnosis, Nosology, Bipolar disorder, Classification

## Abstract

**Background:**

In the 1970 s, scientific research on psychiatric nosology was summarized in Research Diagnostic Criteria (RDC), based solely on empirical data, an important source for the third revision of the official nomenclature of the American Psychiatric Association in 1980, the Diagnostic and Statistical Manual, Third Edition (DSM-III). The intervening years, especially with the fourth edition in 1994, saw a shift to a more overtly “pragmatic” approach to diagnostic definitions, which were constructed for many purposes, with research evidence being only one consideration. The latest editions have been criticized as failing to be useful for research. Biological and clinical research rests on the validity of diagnostic definitions that are supported by firm empirical foundations, but critics note that DSM criteria have failed to prioritize research data in favor of “pragmatic” considerations.

**Results:**

Based on prior work of the International Society for Bipolar Diagnostic Guidelines Task Force, we propose here Clinical Research Diagnostic Criteria for Bipolar Illness (CRDC–BP) for use in research studies, with the hope that these criteria may lead to further refinement of diagnostic definitions for other major mental illnesses in the future. New proposals are provided for mixed states, mood temperaments, and duration of episodes.

**Conclusions:**

A new CRDC could provide guidance toward an empirically-based, scientific psychiatric nosology, and provide an alternative clinical diagnostic approach to the DSM system.

**Supplementary Information:**

The online version contains supplementary material available at 10.1186/s40345-022-00267-3.

## Background

Decades ago, prominent psychiatric researchers proposed Research Diagnostic Criteria (RDC) for all major psychiatric illnesses with the aim of improving the reliability of psychiatric diagnosis (Spitzer et al. 1978). The need now is for new, purely research-based diagnostic criteria aimed primarily at validity (Ghaemi 2018). This paper provides new clinical RDC (CRDC) criteria for bipolar illness.

How we define diagnoses clinically is essential to the success of biological and pharmacological research. When looking for genes for bipolar disorder, or a biological marker, or a pathophysiological change, or mechanisms of drug action, or treatment efficacy, we are constrained by how we define our diagnoses. If the clinical phenotype for bipolar illness is wrong, imprecise or heterogeneous, genetic studies will fail, biological marker studies will be inconsistent, and treatment studies will be ineffective (Ghaemi 2018; Praag et al. 1990).

While validity has to a certain extent been sacrificed to reliability and professional “pragmatism” (Ghaemi 2014), the reverse process may be necessary to achieve validity, since different definitions will have to be tested and some of them may not be easy to use or replicate. Unfortunately, once enshrined, classificatory principles seldom have been questioned and hence four decades of stagnation with respect to identifying causes or treatments for most DSM-defined conditions is a strong argument for seeking a new approach.

If DSM diagnoses are invalid, no matter their reliability, they will not serve as useful tools to identify causes and treatments of mental illness in psychiatric research. For this reason, the United States National Institute of Mental Health leadership has proposed alternative, biologically-based, criteria, the Research Domain Criteria (RDoC), which differentially prioritizes neuroimaging-based findings (Insel et al. 2010). We believe that a clinical research-based approach to diagnostic criteria, which, like the RDoC, is based purely on research evidence, but, unlike the RDoC, is based on *clinical* research, will prove more useful for identifying the causes and treatments of mental illness in psychiatric research.

In this paper we set out the rationale for such a new CRDC for bipolar illness, our field of expertise, and consider the scientific evidence for the validity of the definitions proposed. We hope to demonstrate that this effort can be feasible in a specific condition, like bipolar illness, and that it could then be repeated by experts in other major mental illnesses. This process of developing multiple illness-specific CRDC definitions, similarly focused on clinical research validity, could prove more successful than the purely biologically-based RDoC proposal, as a scientifically-focused research alternative to DSM-based nosologies.

## Methods

The criteria published here largely correspond to the consensus of the ISBD Task Force as published in 2008 (Ghaemi et al. 2008). A narrative review was conducted by members of the task force, subdivided into small groups based on diagnostic subtypes. Members searched the research literature with keywords for their subtype (e.g., “mixed,” “bipolar spectrum,” “bipolar depression”), and supplemented keyword searching with bibliographic cross-referencing. The current proposal adds to the original publication with three major changes, and one minor change. First, since the original Task Force document did not include consensus recommendations for mixed episodes, and since these definitions are meant for research purposes and thus are not definitive, we provide here proposed recommendations for mixed depression based on the two most prominent definitions in recent research, those of Koukopoulos and associates (2007) and Angst and colleagues (2011), along with DSM-5 criteria (defined as types I, II, and III respectively). Second, new definitions of mood temperaments are provided. Third, we have added a criterion to each diagnosis for the duration of the longest episode. This addition is based on evidence that a longer episode may imply greater diagnostic validity than a shorter episode (Angst and Merikangas 2001; Klein 2008).  We have suggested cut-offs between shorter and longer durations of episodes based on past natural history studies, but those suggested cut-offs are preliminary and open to modification with future research. The minor change is that we have replaced the vague and generic term “disorder” with the more specific term “illness”, so as to indicate that bipolar illness is a disease. The term “disorder” was introduced in DSM-III to apply to all diagnoses so as to be “atheoretical” as to etiology; this perspective was meant to reject psychoanalytic theories in particular (Decker 2013). Since few claim psychoanalytic etiologies to bipolar illness, and it is now established that is a mostly genetic disease of the brain and body, as shown in meta-analysis of twin studies (Bienvenu et al. 2011), we need not remain atheoretical in this regard, and, unlike DSM-III to 5, we should be willing to reject a prohibition of terms like “disease” or “illness” when in fact they are present. This relatively minor change in language would be another major advance in putting the past behind us, not continuing to allow the DSM system to inadvertently arrest progress.

## Results

### Relation of manic/hypomanic episodes to subtypes of mood illness

Specific criteria for all diagnoses are provided in the online appendix. As provided in Tables 1, 2, 3, and 4, the proposed definitions of mania (pure or mixed) and hypomania can be used to diagnose bipolar illness of the following subtypes, with the following proposed definitions:

**Table 1:** Validity of CRDC criteria for pure or mixed mania or hypomania

Diagnosis	Symptoms	Course	Family history/Genetics	Treatment effects	Biological
Pure mania	Psychomotor activation along with euphoric mood Low insight and judgment Marked social impairment Less need for sleep May present with irritability/disruptive aggressiveness Marked talkativeness Noticeable flight of ideas Recklessness or increase of sexual drive May present with psychotic features	Usually bipolar, Multiple recurrences alternating with depression (Goodwin and Manic–depressive 2007)	Associated with BP (Goodwin and Manic–depressive 2007)	Responsive to lithium preferentially (Bowden 1995)	Associated with HPA axis activation (Swann et al. 1992) Amygdala enlargement (Foland et al. 2008)
Mixed mania	Psychomotor activation along with dysphoric mood Depressive symptoms usually present Low insight and judgment Marked social impairment Less need for sleep Infrequent rapid cycling Marked talkativeness Noticeable flight of ideas Recklessness Increase of sexual drive May present with psychotic features	Usually bipolar, Multiple recurrences alternating with depression (Goodwin and Manic–depressive 2007)	Associated with BP	Responsive to valproate or antipsychotics preferentially (Bowden 1995; Muralidharan et al. 2013)	Associated with HPA axis activation (Swann et al. 1992) Amygdala, enlargement (Foland et al. 2008)
Pure hypomania	Psychomotor activation along with requirement of euphoric mood Mild if present social impairment Mild decrease of insight and judgment Sleeping decreased Mild talkativeness Mild flight of ideas Mild recklessness or Mild increase of sexual drive	Usually bipolar, alternating with depression (Goodwin and Manic–depressive 2007) Multiple recurrences (Goodwin and Manic–depressive 2007) Highly undetected (Ghaemi et al. 2000)	Associated with BP and MDD to lesser degree (Simpson et al. 1993)	Usually not treated in isolation but may respond to lower levels/doses of mood stabilizers	Little studied
Mixed hypomania	Psychomotor activation along with requirement of dysphoric mood (McElroy et al. 1992) Mild if present social impairment Mild decrease of insight and judgment Sleeping decreased Mild talkativeness Mild flight of ideas Mild recklessness or Mild increase of sexual drive	Usually bipolar, alternating with depression (Suppes et al. 2005) Multiple recurrences (Goodwin and Manic–depressive 2007), (Suppes et al. 2005) Highly undetected (Suppes et al. 2005)	Associated with BP and MDD to lesser degree	Little studied	Unknown

**Table 2:** Validity of CRDC criteria for pure and mixed bipolar depression and bipolar spectrum disorder

Diagnosis	Symptoms	Course	Family history/Genetics	Treatment effects	Biological
Pure depression	Psychomotor slowing along with depressive mood Anhedonia Hyperphagia, hypersomnia Decreasing libido Loss of concentration	Usually bipolar, alternating with mania/hypomania/mixed states (Goodwin and Manic–depressive 2007)	Associated with BP and MDD to lesser degree (Goodwin and Manic–depressive 2007)	Little responsive to antidepressants, unlike MDD, based on placebo–controlled studies (Goodwin and Manic–depressive 2007; McGirr et al. 2016)	Associated with HPA axis activation and hippocampal and prefrontal cortex atrophy (Manji et al. 2001)
Mixed depression type I	Psychomotor activation along with dysphoric mood (Koukopoulos et al. 2007, 2005) Marked inner tension (Koukopoulos et al. 2007, 2005) Marked mood reactivity, rage (Koukopoulos et al. 2007, 2005)	Equally bipolar and unipolar, with recurrent mixed episodes (Koukopoulos et al. 2007, 2005)	Associated with BP (Sani et al. 2014a, b)	Little responsive to antidepressants, more responsive to antipsychotics (Sani et al. 2014a, b)	Unknown
Mixed depression type II	Psychomotor activation along with dysphoric mood Three or more manic symptoms, similar to the DSM–IV approach, along with severe depression, but without any duration criterion (Angst et al. 2011) Irritable mood, flight of ideas, hypersexuality, brief duration	Equally bipolar and unipolar, with recurrent mixed episodes (Angst et al. 2011)	Associated with BP (Angst et al. 2011)	Little responsive to antidepressants, more responsive to antipsychotics (Angst et al. 2011), (Benabarre et al. 2001), (Pae et al. 2012), (Patkar et al. 2012)	Unknown
Bipolar spectrum depression	Psychomotor activation or slowing, with dysphoric mood Severe recurrent depression does not meet classic DSM–III or IV criteria for bipolar disorders type I or type II, nor the classic definition of major depressive disorder (Ghaemi et al. 2002)	Unipolar, with manic symptoms often but not always present (Angst et al. 2018) Highly recurrent (Angst 2007) History of rapid cycling (Angst 2007)	Associated with BP (Angst et al. 2018)	Potentially less responsive to antidepressants, more responsive to antipsychotics or lithium/anticonvulsants (Ghaemi et al. 2002) Frequent presence of antidepressant switch (Ghaemi et al. 2002)	Unknown

**Table 3:** Validity of CRDC criteria for rapid cycling course and pediatric bipolar disorder

Diagnosis	Symptoms	Course	Family history/Genetics	Treatment effects	Biological
Rapid cycling course	Any mood episode	4 or more episodes per year (Goodwin and Manic–depressive 2007)	Associated with BP (Goodwin and Manic–depressive 2007)	Probably worsened by antidepressants, little responsive to any single mood stabilizer, responsive to multiple mood stabilizers (Tondo et al. 2003), (Calabrese et al. 2005), (Ghaemi et al. 2010)	Associated with hypothalamic–pituitary–adrenal axis activation (Juckel et al. 2000)
Pediatric bipolar illness	Episodic changes in energy and mood meeting CRDC definitions for index episodes as above	Bipolar, with depressive episodes Some proportion of cases with onset before 10 years (Soutullo et al. 2005), (Birmaher et al. 2009), (Merikangas et al. 2010)	Associated with BP (Birmaher et al. 2009)	Antipsychotics are effective for acute mania (McClellan et al. 2007) Lithium and anticonvulsants are likely effective for long–term prophylaxis (Kowatch et al. 2005)	Similar patterns as found with adult–onset (Goldstein et al. 2017)

**Table 4:** Validity of CRDC criteria for mood temperaments

Diagnosis	Symptoms	Course	Family history/Genetics	Treatment effects	Biological
Hyperthymic	Persistent mild manic symptoms (Akiskal et al. 1998)	Usually bipolar, alternating with manic and depressive episodes. Sometimes unipolar, alternating with depressive episodes. Sometimes pure and chronic, with no mood episodes (Azorin et al. 2011)	Associated with BP (Vohringer et al. 2012), (Goodwin and Manic–depressive 2007) (Chiaroni et al. 2004)	Little known	Unknown
Cyclothymic	Persistent psychomotor activation alternating with psychomotor slowing, with milder euphoric or irritable mood alternating with dysphoric mood (Akiskal et al. 1998)	Usually bipolar, alternating with manic and depressive episodes. Sometimes unipolar, alternating with depressive episodes. Sometimes pure and chronic, with no mood episodes (Azorin et al. 2011)	Associated with BP (Vohringer et al. 2012), (Goodwin and Manic–depressive 2007), (Chiaroni et al. 2004)	Responsive to low doses/levels of anticonvulsants (Winsberg et al. 2001), (Jacobsen 1993)	Little studied
Dysthymic	Persistent psychomotor slowing along with milder dysphoric mood (Akiskal et al. 1998)	Usually unipolar, alternating with depressive episodes. Sometimes bipolar, alternating with manic–depressive episodes. Sometimes pure, chronic, with no mood episodes (Azorin et al. 2011)	Associated with unipolar depression (Vohringer et al. 2012) (Chiaroni et al. 2004)	Somewhat responsive to antidepressants when unipolar or pure (Levkovitz et al. 2011)	Little studied

*Bipolar illness, type I* is diagnosed when acute manic or mixed manic episodes occur at least once in a lifetime, with depressive episodes frequently also present.

*Bipolar illness, type II* is diagnosed when acute hypomanic episodes occur at least once in a lifetime, with depressive episodes also required.

*Unipolar mania *is diagnosed when acute manic or mixed manic episodes occur at least once in a lifetime, without any history of depressive episodes.

### Validity

There are inevitable differences of interpretation on any scientific matter, and thus the rationale for inclusion of diagnostic criteria as in the tables in this paper will be open to discussion. However, one rationale for the criteria provided is that they represent a consensus of a broad spectrum of researchers in bipolar illness, relying solely on available research data and no other consideration. Besides the relevance of this research-based consensus approach, we provide here the evidence in favor of the validity of the specific criteria provided.

Here we present summary descriptions of the CRDC criteria for bipolar illness, and their validity evidence. Specific criteria are available in an internet-based appendix to this paper.

### Clinical research diagnostic criteria for bipolar illness (CRDC-BP)

#### Pure and mixed mania

The proposed CRDC pure mania definition is largely unchanged from DSM revisions III to 5. The CRDC mixed mania definition derives from recent evidence based on large factor analyses replicated in multiple databases showing that irritable and dysphoric factors are present in manic episodes, such as a cluster analysis of 2179 subjects meeting DSM-IV criteria for an acute manic episode in randomized clinical trials (Swann et al. 2013a). Of this overall sample, the majority met DSM-IV criteria for pure mania (*n* = 1535) versus the DSM-IV mixed definition (*n* = 644). Factor analysis using Young Mania Rating Scale (Young et al. 1978) and Montgomery Asberg Depression Rating Scale (Montgomery and Asberg 1979) items identified five major factors: depression, mania, sleep, insight/poor judgment, and irritability/disruptive aggressiveness. The depression cluster was the largest, capturing 54% of subjects. The mixed manic group was slightly more female (55%) and mostly non-rapid cycling (91%). When stratified by DSM-IV defined pure versus mixed mania, the five clusters remained present to a similar extent, indicating that depressive symptoms are present in manic episodes, whether defined as pure or mixed, according to past DSM definitions. A broadening of the definition for mixed mania, therefore, would be supported by these data, and is represented in the CRDC criteria. A number of other studies in the past two decades also support these findings and conclusions (Cassidy and Carroll 2001; Cassidy et al. 1998; Swann 2000, 2017; Swann et al. 1993; Grunze et al. 2018) (Table 1).

Unlike DSM-IV, but like DSM-5, the CRDC criteria recognize that mixed features are not limited to mania but can also occur with hypomania. In the DSM-5 definition, mania or hypomania can occur with mixed features, if a full manic or hypomanic episode is present along with three or more major depressive features. Also, “MDD with mixed features” is a specifier defined as meeting the criteria for major depression along with the presence of euphoric mood and grandiosity and other manic symptoms. The DSM approach excludes symptoms that “overlap” between depression and mania (irritability, distractibility, psychomotor agitation), thereby excluding the diagnosis of mixed depression when, along with depression, only irritable mood and other manic symptoms are present (Swann et al. 2013b). We see this DSM-5 definition as more reflective of mixed hypomania, rather than mixed depression, since euphoria must be present, a mood state that can alternate with depressed mood but cannot coexist simultaneously with it, unlike irritable mood. In the CRDC criteria, this DSM-5 definition of “MDD or mania/hypomania with mixed features” is used to define “mixed hypomania.” There is limited empirical research on this topic, but some studies suggest that as much as 12% of mood episodes in an unselected bipolar population may be of this mixed hypomanic type as defined here (Koukopoulos and Sani 2014).

#### Pure and mixed hypomania

The CRDC pure hypomania definition is largely unchanged from prior DSM revisions except for the very important question of what should be the defining feature that differentiates hypomania from mania. Traditionally, this has been severity, hypomania being milder in symptoms than mania; this view is reflected in ICD-10 language. In fact, the term “hypomania” dates back to the 1880 s and was used in the psychiatric literature as a synonym for mild mania; Kraepelin wrote: “The slightest forms of manic excitement are called ‘hypomania’…” (Kraepelin 1921) In the 1970 s, Dunner and Fieve suggested hospitalization to be the severity cut off for hypomania versus mania (Fieve et al. 1975). By the time hypomania was included in DSM-IV in 1994, hospitalization was replaced by severe impairment, which is a standard criterion for all DSM diagnoses, and the duration criterion of four days was added arbitrarily, without any empirical evidence, which remains the case still (Parker et al. 2014). Accordingly, these CRDC reduce the duration criterion for hypomania to two days, which does have empirical evidence to support it (Benazzi 2001; Benazzi and Akiskal 2003).

Severe functional impairment also has been seen as present in mania, and absent in hypomania; this is emphasized in the DSM-5 approach. Neither approach is ideal, especially since severity cutoffs are not empirically established, and functional definitions of impairment (e.g., in sexual behavior, or spending money) are influenced by value judgments, which may differ personally and culturally (Moore et al. 1995; Goodwin 2002). The CRDC criteria are based instead on the large psychological literature on cognitive control, which has been suggested to be relevant to mania (Goodwin 2002). That literature involves basic processes of emotion regulation. Extensive neurobiological work has been done, identifying prefrontal cortex and cingulate regions as important in regulation of emotional experience (Green and Malhi 2006; Kjaerstad et al. 2021; Miskowiak et al. 2019; Varo et al. 2021). Aberrant emotion processing underlies abnormal mood and anxiety experiences. Most of this research has focused on depression (Green and Malhi 2006; Kjaerstad et al. 2021; Miskowiak et al. 2019; Varo et al. 2021), but the results are likely relevant also to manic experiences, such as hypomania (Goodwin 2002). This concept of cognitive control, extensively studied in experimental psychology paradigms and in neurobiological work (Green and Malhi 2006; Kjaerstad et al. 2021; Miskowiak et al. 2019; Varo et al. 2021) has more scientific foundation than presumptions about severity or functional impairment. Hence, rather than exclusive reliance on severity or functional impairment, the CRDC criteria have added the key notion that hypomania involves the preservation of such cognitive control over emotional processing, whereas mania does not. Clinically, this cognitive control ability may be perceived as the ability to behave otherwise than one does. Further phenomenological research, correlated with the experimental psychology and neurobiological substrates already established, can help clarify the subjective experience of cognitive control as a clinical feature of hypomania (Table 1).

#### Pure and mixed bipolar depression

An extensive literature demonstrates many features of clinical depression that are more common in bipolar than unipolar depression (Mitchell et al. 2008). These include marked psychomotor slowing, as part of melancholic features of depression (Mitchell et al. 2011; Parker 2007; Parker et al. 2017, 2010). A high recurrence rate of episodes, and other associated features, such as psychotic and “atypical” (hypersomnia, hyperphagia) depressive symptoms, have been demonstrated to be more common in bipolar than unipolar depression (Mitchell et al. 2008). The presence of marked psychomotor slowing and other melancholic features in pure bipolar depression (Parker 2007; Parker et al. 2000) contrasts with the concept of mixed depression, which is seen by investigators as clinical depression associated with psychomotor activation (Koukopoulos et al. 2007; Koukopoulos and Ghaemi 2009). This mixed depression has been conceptualized in different ways, each of which still needs to be further studied to determine which approach is most valid. The CRDC philosophy is not to prejudge such matters, but to identify criteria based on our best available current evidence, and then if there are different approaches, to allow for subtypes with different definitional criteria. Then empirical research can and should be done on each subtype to determine which are valid and which are not (Table 2).

In reviewing this literature, which is mostly limited to the past two decades, we identified three different types of mixed depression as identified and investigated by different researchers. The first subtype, type I, defined by Koukopoulos, describes mixed depression as a state of severe depression with marked inner tension and/or psychomotor agitation, along with marked mood reactivity, rage, and often other manic symptoms (Koukopoulos et al. 2007; Koukopoulos and Ghaemi 2009). This kind of mixed depression can occur, according to these researchers, in both DSM-5 defined MDD and bipolar disorder, but it is especially common in the latter, according to their early empirical studies (Koukopoulos et al. 2007). “Agitated depression” as it has been used in prior research is part of this mixed state concept. The second subtype, type II, defined by Angst, defines mixed depression as three or more manic symptoms, similar to the DSM-5 approach, along with severe depression, but without any duration criterion. Thus mixed depression can involve a few hours to a few days of manic symptoms in the context of a larger depressive episode (Angst et al. 2011). Often these manic symptoms involve irritable mood, flight of ideas, hypersexuality, brief increased psychomotor activity, and similar features. These researchers have conducted a large study in 5635 unselected subjects with a major depressive episode, both MDD and bipolar based on DSM-IV criteria, and found that this definition of mixed depression (termed the “bipolarity specifier”) was present in 47% of that sample (Angst et al. 2011). It was associated with important diagnostic validators: a three-fold increased likelihood of a family history of bipolar illness, and a ten-fold increased odds of antidepressant-induced mania (Angst et al. 2011). Similar findings were observed in the Zurich study cohort of 4547 patients followed for three decades; differences in diagnostic validators of course and genetics were found between depressed patients with manic symptoms (but not full manic episodes) as opposed to patients with pure depressive episodes without any manic symptoms (Angst et al. 2018). A third potential subtype is the DSM-5 definition, which, similar to some research approaches to pediatric mania, requires the presence of euphoric mood and grandiosity, and thus excludes the diagnosis of mixed depression when, along with severe depression, only irritable mood and other manic symptoms are present. This approach excludes symptoms which “overlap” between depression and mania (irritability, distractibility, psychomotor agitation) (Swann et al. 2013b). We see this definition as more reflective of mixed hypomania, rather than mixed depression, since euphoria must be present, a mood state that is incompatible with depression. Thus, in the CRDC criteria, this DSM-5 definition of “MDD or mania/hypomania with mixed features” is located in the hypomania section and termed “mixed hypomania.”

#### Bipolar spectrum depression

Bipolar spectrum depression has been proposed as a clinical construct of unipolar depressive episodes occurring in the context of absence of manic or hypomanic episodes, and not meeting mixed depression criteria above, but associated with other course or genetic or treatment features consistent with bipolar illness. These include a family history of bipolar disorder, antidepressant-induced mania, a highly recurrent course of depressive episodes, brief depressive episodes, and a rapid-cycling unipolar depressive course (Mitchell et al. 2008, 2011; Ghaemi et al. 2002). The basic rationale for this diagnostic grouping is the clinical experience and scientific evidence that many patients with severe recurrent depression do not meet classic DSM-III-5 criteria for bipolar disorders type I or type II, nor the classic definition of major depressive disorder (MDD). In other words, this concept captures the extensive discussion (Akiskal et al. 2002; Akiskal 2002; Akiskal and Pinto 1999; Angst and Cassano 2005; Angst and Gamma 2002; Cassano et al. 1999) and notable literature (Akiskal et al. 2006; Angst 2007, 1998; Angst et al. 1990; Cassano et al. 2004), not only in the past two decades but continuing into current research (Angst et al. 2018; Mazzarini et al. 2018; Mesman and Hillegers 2017), supporting a dimensional spectrum to bipolar illness. For instance, many patients have severe recurrent depressive episodes, but not spontaneous hypomanic or manic episodes, but have parents with bipolar illness, or multiple family members diagnosed with bipolar illness (Akiskal et al. 2003). This observation is consistent with the largest and most validated genome-wide association scans, which definitely find overlap between DSM-defined MDD and bipolar disorder (Grande et al. 2016; Vieta et al. 2018; Sullivan et al. 2012). These findings, now definitive, completely contradict the foundational research in the 1970 s that led to the distinction between MDD and bipolar illness in DSM-III (Gershon et al. 1982). Such cases should be uncommon, but in fact, given the current DSM system with an extremely broad definition of MDD compared to bipolar illness (Ghaemi et al. 2012), these cases are quite common (Smith et al. 2005; Rybakowski et al. 2007) (Table 2).

Further, many patients in clinical practice diagnosed with purported DSM-IV MDD experience mania with antidepressant use. This antidepressant-related mania is rather common in bipolar illness (occurring in about 10–20% of subjects depending on study design and patient characteristics) (Akiskal et al. 2003; Goldberg and Truman 2003), but very uncommon in carefully diagnosed non-bipolar depression (only 0.5% of subjects in the Sequenced Treatment Alternatives for Resistant Depression study) (Perlis et al. 2011). This produces a relative risk of at least 20 (10%/0.5%) that if antidepressant-related mania occurs, the patient does not have unipolar depression; and yet without spontaneous mania or hypomania, the DSM-IV system required the diagnosis of MDD. DSM-5 has dropped this criterion and allows clinicians to fold these subjects into the bipolar type I or type II designations. Some persons only experience mania or hypomania when taking antidepressants, not spontaneously. The bipolar spectrum depression definition would provide a category to capture those patients.

Other criteria somewhat more common in bipolar illness than unipolar depression, like brevity and high recurrence and early age of onset of depressive episodes, are also included in the bipolar spectrum depression definition, but since they have a smaller predictive strength for bipolar illness compared to family history and antidepressant-related mania (Mitchell et al. 2008, 2011; Ghaemi et al. 2002), more of those criteria are required to make this diagnosis.

#### Rapid-cycling course

The CRDC definition provided here differs little from prior DSM revisions. It has been largely validated in nosologic studies, including 20-year prospective data which demonstrate that the four episode cutoff differentiates more severe versus less severe depressive and bipolar illness (Angst and Merikangas 2001). This cutoff should continue to be examined empirically as the number of episodes affect prognosis in a continuous manner (Bauer et al. 2008). There are also important differences in diagnostic validation based on the association of rapid-cycling with course and treatment effects. Most importantly, numerous prospective long-term cohort studies demonstrate that a rapid-cycling course predicts poor prognosis, although rapid-cycling can be transient and return spontaneously to a non-rapid-cycling course (Schneck et al. 2008; Coryell et al. 2003). Randomized clinical trials demonstrate lack of response to any single mood stabilizer (Tondo et al. 2003), whether lithium or an anticonvulsant (Tondo et al. 2003; Calabrese et al. 2005; Ghaemi 2009). Further, the only two randomized studies of the topic find that rapid-cycling predicts worsened long-term course with antidepressant treatment (Ghaemi et al. 2003, 2010) (Table 3).

#### Unipolar mania

Just as some people only experience manic symptoms as part of their temperaments (hyperthymia, see below), without any depressive symptoms, some patients also only experience manic episodes, without any depressive states. DSM-III to 5 legislated away unipolar mania, and mistakenly terms such patients as “bipolar” even though they only have one pole. Past and recent research confirms that some patients exist who only have unipolar manic episodes, without any depressive episodes (Angst and Cassano 2005; Angst et al. 2019; Angst and Grobler 2015; Perugi et al. 2007). There is evidence of differences in prognosis, treatment response, and possibly other clinical features between such unipolar mania and other bipolar subtypes (Angst and Grobler 2015; Perugi et al. 2007). For these patients, the diagnosis of unipolar manic illness would be appropriate.

#### Pediatric bipolar illness

The validity of pediatric bipolar illness has been very controversial, especially when focusing on impulsivity, anger, and affective lability. DSM-5 took the approach of using the same criteria for diagnosing bipolar disorder in children and adolescents as in adults (with the exception of shorter duration for cyclothymic disorder), emphasizing that bipolar illness is rare before puberty, and creating a new diagnosis for chronically irritable and angry children (disruptive mood dysregulation disorder). We advocate focusing on whether and how clinicians can make a bipolar diagnosis in children, rather than focusing on unclear or vague cases characterized by anger and affective lability (Evans et al. 2017) (Table 3).

There are a number of lines of evidence demonstrating the existence of bipolar illness in children. These have been reviewed in the ISBD Child Task Force papers in 2008 (Youngstrom et al. 2008) and updated later (Goldstein et al. 2017). Meta-analyses of pediatric epidemiological samples find that bipolar illness occurs in children and adolescents at similar rates around the world, with rates increasing in adolescence. Longitudinal data show developmental continuity and similar course as bipolar identified in adults (Axelson et al. 2015, 2011). Imaging, genetics, clinical characteristics and treatment response all show congruence between pediatric and adult cases (Youngstrom et al. 2008; Goldstein et al. 2017; McClellan et al. 2007). Many, though not all, studies find evidence of prepubertal mania in at least some children. Age of onset studies find that about 10% of the reported onset of bipolar illness in the National Comorbidity Study occurred before age 12 (Kessler et al. 2005). Direct studies of children below age 12 identify subjects who meet DSM-IV criteria of mania in up to 4% of children seen in psychiatric clinic settings (Soutullo et al. 2005; Birmaher et al. 2009). Extensive data now show the validity of checklists as aids for identification of bipolar illness in youths (Youngstrom et al. 2015) as well as adults (Youngstrom et al. 2018). Despite these findings, some clinicians strongly oppose the diagnosis of bipolar illness, or mania, below age 12, and others insist on a narrow diagnosis using only euphoric mood and presence of grandiose ideation (Geller et al. 2000), as opposed to the standard adult criteria for mania which include irritable mood and do not require presence of grandiose ideation. Given the data on acute mania reviewed above (Swann et al. 2013a), indicating that about 54% of manic episodes involved the presence of depressed, not elated, mood, it would seem unsupported by scientific evidence to narrow the diagnosis of mania in children to only elated mood. Further, it would seem even more unsupported to refuse that diagnosis to any children when well-conducted population-based studies identify the presence of mania in adolescence (Merikangas et al. 2010). Finally, even studies in preadolescent children identify presence of mania in replicated settings in many different countries, cultures, and professional settings (Soutullo et al. 2005). On these grounds, the CRDC criteria define the presence of pediatric mania using the same criteria as are applied for adult mania, consistent with ICD-11 and DSM-5. The definition of the phrase “pediatric” is the standard definition of non-adulthood, namely below age 18.

#### Temperaments

The concept of temperaments has been included for two of three mood temperaments (dysthymia and cyclothymia) in prior DSM revisions, but they have been listed as separate from personality conditions. The three mood temperaments in the CRDC definitions (including hyperthymia) have been defined since Kraepelin’s textbook revisions dating at least to 1921 and to earlier editions with different names (“constitutional excitement”, “constitutional depression”) (Kraepelin 1921). Other studies over two decades demonstrate validity of these constructs based on genetic (Kelsoe 2003; Vohringer et al. 2012) and course (Vohringer et al. 2012; Akiskal 2007) differences compared to other subtypes of bipolar illness and compared to personality disorders (Pompili et al. 2018). For instance, dysthymia and cyclothymia are more common in family members of persons with bipolar illness compared to family members of normal controls (Evans et al. 2005). Mild manic symptoms are more strongly linked in family studies to probands with bipolar illness type I, rather than similar severe cases of mania (type I bipolar disorder) in family members (Simpson et al. 1993; Hantouche and Akiskal 2006). The most common mood temperament among patients with bipolar illness appears to be cyclothymia, present in 40% of patients in one study, distantly followed by hyperthymia and dysthymia in less than 15% (Vohringer et al. 2012). In that study, about one-half of patients with bipolar illness did not meet the definition of any mood temperament, so although these temperaments are common in bipolar illness, they are not present in all patients (Vohringer et al. 2012). Cyclothymia and hyperthymia, although characterized by mood reactivity, like borderline personality, are also associated genetically with bipolar illness (Akiskal et al. 1977), unlike borderline personality, for which genetic evidence is inconsistent and mostly indicative of low heritability (Bienvenu et al. 2011; Bassett 2012) Further, the specific symptom definitions of these temperaments goes far beyond mere mood reactivity, and thus include many features not found in borderline or other personality disorders (Bassett 2012; Barroilhet et al. 2013; Ghaemi et al. 2014). Lastly, specific association with self-cutting and past sexual abuse, central to the concept of borderline personality (Bassett 2012; Gunderson 1984), is not prevalent to any notable degree in these mood temperaments, based on empirical course studies (Barroilhet et al. 2013; Ghaemi et al. 2014) (Table 4).

## Discussion

In 2008, the International Society for Bipolar Disorders Diagnostic Guidelines Task Force provided recommendations for the diagnosis of bipolar disorder (Ghaemi et al. 2008). These recommendations were meant for both clinicians and researchers, and as input to the DSM-5 process. Five years later, in 2013, DSM-5 was published, with the inclusion of some ISBD Task Force members, but most of the recommendations of the task force were ignored.

DSM-5 proposes three main changes in the diagnostic criteria for bipolar illness: including abnormally and persistently increased activity or energy as a core feature of (hypo)mania along with elevated mood; removal of the exclusion for antidepressant-induced mania; and removal of the mixed episode syndrome, replaced by the addition of mixed symptoms as a modifier for hypomanic, manic and depressive episodes, not only in bipolar illness but also in major depressive disorder (MDD) (www.dsm5.org). These revisions are consistent with the recommendations of the ISBD task force. They appear to decrease the point prevalence of bipolar illness (Kessing et al. 2021). Other recommendations made by the ISBD task force but not incorporated in the DSM-5 proposal address the following: pediatric bipolar disorder, hypomania duration, schizoaffective disorder, bipolar depression, and the concept of a bipolar spectrum. 

The DSM approach to bipolar illness is Leonhardian, not Kraepelinian. DSM took the approach of Karl Leonhard, dividing manic-depressive illness (MDI) into two categories, bipolar and unipolar. Kraepelin held that MDI was a broad spectrum of one illness (Goodwin and Jamison 2007). This paper  does not address unipolar depression, but it provides diagnostic validity evidence from the last three decades of research that tends to oppose the radical DSM-III decision in 1980 to divide MDI and to create a very narrow bipolar definition in the Leonhardian tradition.

Many clinicians will continue to defend DSM definitions as based on empirical evidence. This matter is addressed in more detail elsewhere showing the very limited amount of empirical evidence used in DSM revisions, in contrast to the admitted approach of DSM leaders (Frances et al. 2013) that “pragmatic” non-scientific considerations are the most important. An example relevant to bipolar illness is the four day criterion for hypomania, created arbitrarily in 1994 by DSM-IV. There is no empirical evidence at all to support that cut-off, as the ISBD task force documented (Ghaemi et al. 2008). The purpose of the CRDC is to base criteria solely on empirical evidence, and nothing else.

Defenders of DSM revisions will emphasize that it has reliability, even if its validity may be questioned. In fact, even the reliability of the DSM system is weak. Given that a kappa of 0.5 indicates that observers agree and disagree equally frequently, statistical experts have recommended a minimum of 0.6 as acceptable kappa (McHugh 2012). On that standard, the DSM-5 field trials only had acceptable reliability for alcohol use disorder among major diagnoses. All other kappas fell below 0.6, including bipolar disorder (kappa = 0.56), and most were below 0.4 (Regier et al. 2013). In fact, the reliability of MDD was quite poor (kappa was only 0.23) and had declined markedly in 2013 compared to its nearly identical definition with DSM-III in 1980. Hence the DSM-III distinction between bipolar disorder and MDD not only may be false in validity but it has very poor reliability too.

It might be suggested that these broader definitions of bipolar illness could have harmful clinical effects since their predictive value is unknown, and false positives may be seen in cases where patients might have “major depressive disorder” (MDD) instead. Since MDD often responds to psychotherapies alone, especially cognitive behavioral therapy, whereas the full spectrum of bipolar illness (including mood temperaments) often requires some kind of medication treatment, then such misdiagnosis might lead to overuse of medications. This possibility must be interpreted in the context of a few factors. First, the diagnostic validity of “MDD” has never been proven, and in fact is highly questionable, as the concept was socially constructed in DSM-III in 1980, and hardly revised since (Decker 2013; Ghaemi 2013). It involved a fusion of the concept of neurotic depression, which involved persons with mild to moderate depression and anxiety without genetic diathesis (Ghaemi 2008; Shorter 2007), with subjects with unipolar manic-depressive disease, which involved severe recurrent depressive episodes that lasted 6–12 months or longer with strong genetic diathesis (Shorter 2007). Further, non-recurrent chronic severe depression, previously called “involutional melancholia” also was combined into the MDD construct. Hence, the notion that the DSM-based heterogenous MDD construct has scientific validity itself is questionable (Decker 2013; Ghaemi 2013). The pre-DSM-III concept of manic-depressive illness (MDI) meant not only bipolar illness but also unipolar depression; polarity did not matter; the key definition of the illness was recurrent mood episodes of any kind (Kraepelin 1921; Goodwin and Jamison 2007; Ghaemi and Dalley 2014). This broad definition of MDI has not been refuted based on research over the last half century; it was simply rejected in 1980 by DSM-III and its consequences have been accepted uncritically (Ghaemi and Dalley 2014).

Hence, the common criticism that broad definitions of bipolar illness are harmful ignores the equally valid criticism that broad definitions of MDD are harmful. The key to solving these dilemmas is to base all diagnostic definitions on clinical research solely, not on social or economic or professional preferences. This absolute research-based philosophy is the key difference between these CRDC and DSM-5, taking into account that research data may be limited on some topics, in which case the research-based approach would be to use the best research data available at the time, or to refrain from providing criteria where no research data exist.

### Future possibilities

This paper seeks to systematize our current nosology of bipolar illness more fully and empirically than is the case with the DSM system. It starts with, and is reliant upon, currently available data. Future data could direct these CRDC in new directions, some of which might be hypothesized here. A non-exhaustive list of questions for further evaluation follow:

1. Is the bipolar/unipolar distinction itself legitimate, or should we go back to some version of the broader manic-depressive illness concept?
2. Within the bipolar concept, is the type I versus type II distinction legitimate, or should it be viewed as invalid nosologically, or mainly important therapeutically?
3. Is the distinction between hypomania and mania legitimate? Just as there is no distinction between “hypodepression” and depression, is this terminology based on severity of illness diagnostically valid or needed?
4. Is the definition of rapid-cycling based on four or more episodes in a year legitimate? This definition is based on observational data from the 1970s, and its validity as opposed to other possible definitions has not been explored.
5. Should mixed states be defined in the context of acute mood states, as they are at present (i.e. a depressive episode with manic symptoms, or a manic episode with depressive symptoms)? Does this approach emphasize polarity excessively?

## Conclusions

All revisions of DSM since 1980 have suffered from an overly pragmatic approach to diagnostic definitions. Those definitions have been constructed for many purposes, with research evidence not being primary. Unfortunately, DSM-5 has not rectified this problem. Whatever the professional and social benefits of such pragmatic judgments, biological and clinical research needs to rely on diagnostic definitions based as far as possible on research evidence solely. To that end, we propose here Clinical Research Diagnostic Criteria for Bipolar Illness for use in research studies, with the hope that these criteria may lead to further refinement of diagnostic definitions in the future for all other major mental illnesses. Unlike a heavily neurobiological RDoC proposal, systematic CRDC definitions will provide a *clinically-based* approach towards a scientific psychiatric nosology. A positive outcome would be if these CRDC criteria were used by the profession in general to study different diagnostic approaches to bipolar illness, so as to be further validated or or revised or refuted based on those studies. Researchers then could revise the CRDC criteria every few years based on emerging studies, providing more accurate and more rapid progress diagnostically than has been the case since DSM-III.

## Supplementary information

**Additional file 1. Online Supplementary Appendix: Clinical Research Diagnostic Criteria for BipolarDisorder.**

## Data Availability

No new databases included. Data sharing statement: Data sharing is not applicable to this article as no new data were created or analyzed in this study.

## Abbreviations

BP: Bipolar
DSM: Diagnostic and Statistical Manual
ICD: International Classification of Diseases
ISBD: International Society for Bipolar Disorders
MDD: Major depressive disorder
CRDC: Clinical Research Diagnostic Criteria
RDoC: Research Domain Criteria
