# Supplementary material for: Clinical research diagnostic criteria for bipolar illness (CRDC-BP): rationale and validity
Source: Int J Bipolar Disord. 2022 Oct 13;10:23. doi: 10.1186/s40345-022-00267-3 (PMC9561456; doi:10.1186/s40345-022-00267-3)
Supplement: Supplementary file 1 — Additional file 1. Online Supplementary Appendix: Clinical Research Diagnostic Criteria for BipolarDisorder. [file 40345_2022_267_MOESM1_ESM.docx]

**Appendices. Research Diagnostic Criteria for Bipolar Illness (RDC-BP)**

Appendix 1. RDC for acute pure and mixed mania

1. A distinct period of persistently elevated, expansive, or irritable mood, lasting throughout at least 7 days, that is clearly different from the usual non depressed mood.
2. During the period of mood disturbance, three (or more) of the following symptoms have persisted (four if the mood is only irritable) and have been present to a significant degree:
   1. inflated self-esteem or grandiosity
   2. decreased need for sleep (e.g., feels rested after only 3 hours of sleep)
   3. more talkative than usual or pressure to keep talking
   4. flight of ideas or subjective experience that thoughts are racing
   5. distractibility (i.e., attention too easily drawn to unimportant or irrelevant external stimuli)
   6. increase in goal-directed activity (either socially, at work or school, or sexually) or psychomotor agitation
   7. excessive involvement in pleasurable activities that have a high potential for painful consequences (e.g., the person engages in unrestrained buying sprees, sexual indiscretions, or foolish business investments)
3. *Increased activity or energy is present, along with the above two criteria*
4. The episode is severe enough to cause marked impairment in social or occupational functioning, or to necessitate hospitalization
5. *The symptoms are not due to a general medical condition*
6. *State duration of longest episode: Differentiate in particular less than 2 weeks versus greater than 2 weeks.*

***Mixed mania***

*A. Along with a manic episode, presence of two or more of the following:*

## *1. Depressed mood*

## *2. Anhedonia*

## *3. Fatigue or loss of energy*

## *4. Excessive or inappropriate guilt*

## *5. Recurrent thoughts of death or suicidal ideation*

## *6. Severe anxiety*

*7. Marked irritability*

# *B. The symptoms cause clinically significant distress or impairment in social or occupational functioning.*

*C. The symptoms are not due to a general medical condition*

*D. State duration of longest episode: Differentiate in particular less than 2 weeks versus greater than 2 weeks.*

### Appendix 2. RDC for pure and mixed hypomania

*Pure hypomania*

A. A distinct period of persistently elevated, expansive, or irritable mood, lasting throughout at least 2 days, that is clearly different from the usual non depressed mood.

B. During the period of mood disturbance, three (or more) of the following symptoms have persisted (four if the mood is only irritable) and have been present to a significant degree:

- 1. inflated self-esteem or grandiosity
  2. decreased need for sleep (e.g., feels rested after only 3 hours of sleep)
  3. more talkative than usual or pressure to keep talking
  4. flight of ideas or subjective experience that thoughts are racing
  5. distractibility (i.e., attention too easily drawn to unimportant or irrelevant external stimuli)
  6. increase in goal-directed activity (either socially, at work or school, or sexually) or psychomotor agitation
  7. excessive involvement in pleasurable activities that have a high potential for painful consequences (e.g., the person engages in unrestrained buying sprees, sexual indiscretions, or foolish business investments)

C. Increased activity or energy is present, along with the above two criteria

D. The episode is associated with an unequivocal change in functioning that is uncharacteristic of the person when not symptomatic.

E. The disturbance in mood and the change in functioning are observable by others*.*

F. The episode is not severe enough to cause marked impairment in social or occupational functioning, or to necessitate hospitalization, and there are no psychotic features, although mild-to-moderate depressive symptoms may be present (mixed hypomania).

1. Cognitive control - defined as the patient’s ability to express their emotions in ways that are personally and socially appropriate , as judged by the clinician and others like family members, during the period of mood elevation - is largely intact.
2. The symptoms are not due to a general medical condition or directly attributable to a drug of abuse
3. State duration of longest episode: Differentiate in particular less than 2 weeks versus greater than 2 weeks.

*Mixed hypomania (equivalent to proposed DSM-5 criteria for major depressive disorder with mixed features)*

A Full criteria are met for a Major Depressive Episode, along with at least 3 of the following symptoms present nearly every day during the episode.

1. Elevated, expansive mood

2. Inflated self-esteem or grandiosity

3. More talkative than usual or pressure to keep talking

4. Flight of ideas or subjective experience that thoughts are racing

5. Increase in energy or goal directed activity (either socially, at work or school, or sexually)

6. Increased or excessive involvement in activities that have a high potential for painful consequences (e.g., engaging in unrestrained buying sprees, sexual indiscretions, or foolish business investments).

7. Decreased need for sleep (feeling rested despite sleeping less than usual; to be contrasted from insomnia)

C.   Mixed symptoms are observable by others and represent a change from the person’s usual behavior.

C. Manic symptoms cause clinically significant distress or impairment in social, occupational, or other important areas of functioning; or they represent an unequivocal change from usual behavior

D. The symptoms are not due to a general medical illness or directly attributable to a drug of abuse.

E. The symptoms persist for 2 weeks or longer, as in a major depressive episode

E. State duration of longest episode: Differentiate in particular less than 4 weeks versus greater than 4 weeks.

Appendix 3. Research diagnostic criteria for pure bipolar depression

A. A past manic or hypomanic episode

B. A major depressive episode characterized by five (or more) of the following symptoms have been present during the same 2-week period and represent a change from previous functioning; at least one of the symptoms is either 
   (1) depressed mood or
   (2) loss of interest or pleasure.

(1) depressed mood most of the day, nearly every day, as indicated by either subjective report (e.g., feels sad or empty) or observation made by others (e.g., appears tearful).
(2) markedly diminished interest or pleasure in all, or almost all, activities most of the day, nearly every day (as indicated by either subjective account or observation made by others) 
(3) significant weight loss when not dieting or weight gain (e.g., a change of more than 5% of body weight in a month), or decrease or increase in appetite nearly every day.
(4) hypersomnia or insomnia nearly every day 
(5) psychomotor retardation or agitation nearly every day (observable by others, not merely subjective feelings of restlessness or being slowed down) 
(6) fatigue or loss of energy nearly every day 
(7) feelings of worthlessness or excessive or inappropriate guilt (which may be delusional) nearly every day (not merely self-reproach or guilt about being sick) 
(8) diminished ability to think or concentrate, or indecisiveness, nearly every day (either by subjective account or as observed by others) 
(9) recurrent thoughts of death (not just fear of dying), recurrent suicidal ideation without a specific plan, or a suicide attempt or a specific plan for committing suicide

C. The symptoms cause clinically significant distress or impairment in social, occupational, or other important areas of functioning.

D. The symptoms are not due to the direct physiological effects of a substance (e.g., a drug of abuse, a medication) or a general medical condition (e.g., hypothyroidism).

E. Special consideration should be given to the presence of symptoms indicative of bipolar depression: (a) melancholic depressive symptoms (mood non-reactivity, marked psychomotor retardation, diurnal mood variation worse in the morning, marked anhedonia), (b) atypical features: increased sleep and/or appetite, (c) psychotic features, (d) early age of onset of depression (< 20 years), (e) a highly recurrent course (>5 episodes), (f) brief major depressive episodes (< 3 months in duration), (g) a positive family history of bipolar disorder.

F. State duration of longest episode: Differentiate in particular less than 4 weeks versus greater than 4 weeks.

Appendix 4. Research diagnostic criteria for mixed depression

*Type I (proposed by Koukopoulos)*

A. Along with a major depressive episode, presence of three or more of the following:

1. Presence of psychomotor agitation (and absence of psychomotor retardation)

2. Marked irritability or unprovoked rage

3. Marked mood lability/reactivity

4. Psychic agitation/inner tension/severe anxiety

5. Talkativeness

6. Accelerated thinking (irrespective of content, which could be anxious, depressive, or indifferent or neutral)

7. Dramatic description of suffering and/or frequent spells of weeping

8. Insomnia (not hypersomnia)

9. Occasionally increased libido or hypersexuality

B. The symptoms do not meet criteria for a manic episode.

C. The symptoms cause clinically significant distress or impairment in social, occupational, or other important areas of functioning.

D. The symptoms are not due to a general medical illness or directly attributable to a drug of abuse.

E. The symptoms persist for 2 weeks or longer, as in a major depressive episode

F. State duration of longest episode: Differentiate in particular less than 4 weeks versus greater than 4 weeks.

*Type II (proposed by Angst and Benazzi)*

A. Along with a major depressive episode, presence of three or more manic symptoms

1. Distractibility

2. Inflated self-esteem or grandiosity

3. More talkative than usual or pressure to keep talking

4. Flight of ideas or subjective experience that thoughts are racing

5. Increase in energy or goal directed activity (either socially, at work or school, or sexually)

6. Increased or excessive involvement in activities that have a high potential for painful consequences (e.g., engaging in unrestrained buying sprees, sexual indiscretions, or foolish business investments).

7. Decreased need for sleep (feeling rested despite sleeping less than usual; to be contrasted from insomnia)

B. Manic symptoms do not meet duration criteria for a hypomanic episode, i.e. two days or more

C. Manic symptoms cause clinically significant distress or impairment in social, occupational, or other important areas of functioning; or they represent an unequivocal change from usual behavior

D. The symptoms are not due to a general medical illness or directly attributable to a drug of abuse.

E. The symptoms persist for 2 weeks or longer, as in a major depressive episode

F. State duration of longest episode: Differentiate in particular less than 4 weeks versus greater than 4 weeks.

Appendix 5. Research diagnostic criteria for bipolar spectrum illness

1. Presence of Recurrent Major Depressive Episodes (3 or more episodes)

2. Absence of Hypomanic or Manic Episodes

3. Presence of multiple signs of bipolarity from three of the following four categories:

a. Treatment effects (nonresponse to three or more therapeutic antidepressant trials, antidepressant-induced syndromal acute mania/hypomania, antidepressant-related long-term mood destabilization, antidepressant-induced irritability, antidepressant-induced suicidality). Note: antidepressant effects need to be clinically distinguishable from akathisia.

b. Family history (assessed in first and second degree relatives: bipolar diagnoses; multi-generational mental illness; alcohol and other substance use; suicides)

c. Depressive symptom phenomenology (melancholic, seasonal, psychotic)

d. Course of illness (early age of onset <20 years, short duration of episode < 3 months, highly recurrent course with > 5 episodes)

4.State duration of longest depressive episode: Differentiate in particular less than 4 weeks versus greater than 4 weeks.

Appendix 6. Research diagnostic criteria for rapid-cycling course

Rapid cycling (can be applied to Bipolar I Illness, Bipolar II Illness, or Bipolar Spectrum Illness):

A. At least four fully syndromal episodes of a mood disturbance in the previous 12 months that meet criteria for a major depressive, mixed depressive (types I or II or III), manic, or hypomanic episodes.

B. Episodes are demarcated either by partial or full remission or a switch to an episode of opposite polarity (e.g., Major Depressive Episode to Manic Episode).

C. State duration of longest episode: Differentiate in particular less than 2 weeks versus greater than 2 weeks.

D. Specify duration of interepisodic interval: Differentiate in particular less than 2 weeks versus greater than 2 weeks.

E. Specify Continuous Rapid-Cycling if there is no interepisode interval at all

Appendix 7. Research diagnostic criteria for pediatric bipolar illness

1. Presence of acute manic or hypomanic syndromal mood episodes, plus depressive episode, prior to age 18.
2. These mood episodes are above and beyond what is expected for the developmental state of the child, and clearly not secondary to other medical or psychiatric disorders or environmental conditions
3. The definition of the acute manic or hypomanic or mixed episode meets adult criteria
4. Manic or hypomanic episodes must be distinct changes from baseline non-manic or hypomanic-mood states.
5. State duration of longest episode: Differentiate in particular less than 2 weeks versus greater than 2 weeks.

Appendix 8. Research diagnostic criteria for mood temperaments

*Hyperthymia*

**A.** Mildly elevated mood for most of the day, for more days than not, as indicated either by subjective account or observation by others, for most of a person’s lifetime.

**B.** Presence, while with elevated mood, of three (or more) of the following:

(1) Being highly extroverted: high interpersonal warmth and people-seeking
(2) Cheerfulness: being overoptimistic or exuberant, often very humorous
(3) Being highly open to experience: Stimulus-seeking, seeking new projects, having expertise or ability in many areas; can reflect impulsive behavior, such as excessive spending
(4) Over-involvedness in other’s activities or lives: sometimes can be meddlesome
(5) A high amount of goal-directed activity: Being always on the go, a “workaholic”; can be reflected sometimes in improvident or impulsive decision-making

(6) Being full of ideas: Constant rapid accelerated thinking, planning numerous projects or activities at once
(7) Overconfidence: Being very self-assured, boastful, bombastic or even sometimes seeming entitled or grandiose

(8) Having a generally high sexual drive; can involve sexual promiscuity

(9) Being a habitual short-sleeper: normally needing less than 6 hours nightly without feeling tired

**C.** During most of a person’s lifetime, the person has never been without the [symptoms](http://behavenet.com/symptom) in Criteria A and B for more than 2 months at a time.

**D.** The above condition precedes and follows mood episodes; it does not only begin after mood episodes. In other words, it is not better accounted for by chronic Manic/Hypomanic or Major Depressive Disorder, In Partial Remission. 
Note:  There may be superimposed episodes of Mania or Major Depressive Disorder, in which case both diagnoses may be given when the criteria are met for a Manic or Major Depressive Episode. (Hypomanic episode criteria are not met because hyperthymia is by definition not episodic).

*Dysthymia*

**A.** Mildly depressed mood for most of the day, for more days than not, as indicated either by subjective account or observation by others, most of a person’s lifetime.

**B.** Presence, while depressed in mood, of three (or more) of the following:

(1) Being gloomy or somber  
(2) Being incapable of fun, even joyless
(3) Being brooding and pessimistic
(4) Low-self esteem
(5) Preoccupation with failure 
(6) Being self-critical

(7) Being unenergetic, sluggish, lacking drive

(8) Being bound to routine

(9) Being quiet and taciturn

(10) Being very shy

**C.** For most of person’s lifetime, the person has never been without the [symptoms](http://behavenet.com/symptom) in Criteria A and B for more than 2 months at a time.

**D.** The above condition precedes and follows mood episodes; it does not only begin after mood episodes. In other words, it is not better accounted for by chronic Manic/Hypomanic or Major Depressive Disorder, In Partial Remission. 
Note: There may be superimposed episodes of Mania/hypomania or Major Depressive Disorder, in which case both diagnoses may be given when the criteria are met for a Manic/hypomanic or Major Depressive Episode.

*Cyclothymia*

**A.** Mildly elevated mood for most of the day, alternating with mildly depressed mood, for more days than not, as indicated either by subjective account or observation by others, for most of a person’s lifetime.

**B.** Presence while depressed or elevated in mood, of three (or more) of the following:

(1) Rapid shifts in mood or energy
(2) Mood or energy up or down, rarely in between
(3) Thinking ability fluctuates greatly
(4) Alternating between bubbling with energy and being sluggish
(5) Perceptions vary between vivid and dull 
(6) Alternating between being sociable and withdrawn

(7) Mood changes without knowing why

(8) Enjoying people or activities and then losing interest

(9) Experiencing all emotions intensely

**C.** For most of a person’s lifetime, the person has never been without the [symptoms](http://behavenet.com/symptom) in Criteria A and B for more than 2 months at a time.

**D.**The above condition precedes and follows mood episodes; it does not only begin after mood episodes. In other words, it is not better accounted for by chronic Manic/Hypomanic or Major Depressive Disorder, In Partial Remission. 
Note:  There may be superimposed episodes of Mania/hypomania or Major Depressive Disorder, in which case both diagnoses may be given when the criteria are met for a Manic/hypomanic or Major Depressive Episode.
